# Effects of aging and exercise habits on blood flow profile of the ocular circulation

**DOI:** 10.1371/journal.pone.0266684

**Published:** 2022-04-14

**Authors:** Chihyun Liu, Tatsuhiko Kobayashi, Tomoaki Shiba, Naoyuki Hayashi

**Affiliations:** 1 School of Environment and Society, Department of Social and Human Sciences, Tokyo Institute of Technology, Tokyo, Japan; 2 Department of Ophthalmology, School of Medicine Toho University, Tokyo, Japan; 3 Department of Ophthalmology, International University of Health and Welfare, Narita Hospital, Chiba, Japan; 4 Faculty of Sport Sciences, Waseda University, Saitama, Japan; National Institutes of Health, National institute of Diabetes and Digestive and Kidney Diseases, UNITED STATES

## Abstract

**Purpose:**

We examined the effects of aging and exercise habits on the ocular blood flow (OBF) and its profiles throughout the optic nerve head region and choroidal area. We hypothesized that exercise habits reduce the stiffness of vessels in the ocular circulation, which generally increases with aging.

**Methods:**

Participants in a medical checkup program (698 males and 192 females aged 28 to 80 years) were categorized into 2 groups (with and without exercise habits) based on participant self-reporting and the definition of the Ministry of Health, Labor and Welfare of Japan (MHLW). OBF in the right eye was measured and analyzed using laser speckle flowgraphy. The blowout time (BOT), which is the time during which the blood flow is higher than half of the mean of the minimum and maximum signals during one heartbeat, was calculated as an index of the blood flow profile. BOT has been used as an indicator of the flexibility of blood vessels.

**Results:**

BOT significantly decreased with aging. Neither the self-reported nor MHLW-based exercise habits significantly affected the ocular circulation.

**Conclusion:**

These results indicate that the stiffness of the ocular vessels increases with aging, and this cannot be prevented by exercise habits.

## Introduction

There will be considerable growth in the aging population during the coming decades. Aging results in structural and functional impairments in ocular vascular networks [1], and consequently increases the risk of vision impairment [2], especially among those older than 50 years [3]. In countries with aging societies such as Japan, eye diseases such as diabetic retinopathy, glaucoma, and age-related macular degeneration are relatively common. Preventing ocular vascular dysfunction can improve the quality of life.

Physical exercise habits may benefit people with visual diseases [4]. People who engage in physical activity had lower rates of glaucomatous vision loss [5]. Another correlational study analyzed over 8000 children’s data in Ireland, revealing that refractive error and vision problems were significantly associated with increased sedentary behavior and decreased physical activity [6]. Nevertheless, effects of exercise habits on preventing ocular vascular dysfunction is still unclear.

Exercise habits have been shown to improve vascular stiffness not only in the limbs but also many other regions of the body [7], whereas effects of exercise habits on the ocular circulation remain unknown Effects of exercise habits on improving vascular stiffness can vary between individuals and body [8]. Exercise habits are strongly recommended, but a large proportion of adults cannot meet these recommendations, including in Japan [9]. This situation indicates the importance of research into effect of exercise habits on ocular circulation. Thus, the present cross-sectional study examined the effects of aging and exercise habits on the ocular blood flow (OBF) and its profiles (i.e., indices of ocular vascular flexibility) in the optic nerve head (ONH) and choroidal regions in healthy participants covering a wide age range. We hypothesized that the stiffness of the ocular circulation increases with aging and that exercise habits can decrease the stiffness, because they have been shown to be effective at many other sites [10].

## Methods

The present study was approved by the Research Ethics Committee for Tokyo Institute of Technology (approval number 2018036). All of the protocols used conformed with the standards set by the Declaration of Helsinki. Each participant received verbal and written explanations of the objectives, measurements, and risks and benefits associated with this study, after which written informed consent was obtained.

### Participants and study protocol

We used the data obtained from 1,079 participants in the medical checkup program at the Department of Health Care Center of the Japan Community Health Care Organization, Tokyo Kamata Medical Center. Participants who had metabolic syndrome as identified based on the Japanese committee to Evaluate Diagnostic Standards for Metabolic Syndrome were included [11, 12]. Participants were excluded if they had an ophthalmic disease, such as glaucoma, uveitis, optic neuropathy, vitreous or retinal disease, or retinal and choroidal vascular diseases; or atherosclerotic diseases such as hypertension, dyslipidemia, diabetes mellitus, cardiovascular or cerebrovascular events, arrhythmia, or exclusion criteria and a best-corrected visual acuity of <40/50; or if they had undergone a previous intraocular surgery. And we had also confirmed the medical history of the drug by inquiry and the eye disease by the photograph of the fundus.

The study criteria were finally met by 880 participants (698 males and 182 females), and the measurements were made from December 2016 to December 2018. And participants who had metabolic syndrome were met by 177 participants (172 males and 5 females).

The participants were categorized into three groups according to their age: young (<40 years), middle-aged (41–64 years), and elderly (>65 years). The participants were also categorized into two groups according to their exercise habits (Ex group and Non-ex group) as determined based on two criteria: (1) self-reporting in a questionnaire and (2) the definition of the Ministry of Health, Labor and Welfare of Japan (MHLW). In the MHLW definition, exercise habits are present when exercising more than twice a week for more than 30 minutes at a time and for more than 1 year.

The participants were instructed to not consume caffeine-containing or alcohol-containing beverages or spicy food, and to not perform high-intensity exercise for at least 1 day prior to the measurements. The participants were also instructed to not smoke on the experimental day. All of the evaluations were performed between 0900 and 1100 hours, after the participants had fasted overnight. Participants were allowed to wear contact lens but not glasses during the OBF measurement, with pupil-dilating eye drops not being used. Excellent repeatability was previously demonstrated between the mean blur rate (MBR) measurements made with and without pupil dilation [13].

### Measurements

#### Ocular circulation

The participants first rested for 10 minutes in a room at a temperature of 24°C. The face was then fixed on a measurement base, and the blood flow profile in the right eye was measured for 4 seconds in the seated position using laser speckle flowgraphy (LSFG).

#### Laser speckle flowgraphy

LSFG was applied using a system (LSFG-NAVI, Softcare, Fukuoka, Japan) developed to measure OBF and assess the OBF profile [14]. LSFG assesses Mean Blur Rate (MBR), which reflects the relative blood flow velocity and is correlated with the actual blood flow volume as measured using hydrogen gas clearance and microsphere methods [15].

We assessed the blood flow profile using LSFG Analyzer software (version 3.0.47, Softcare). The detail of the analysis was similar to those reported previously [16, 17]. The software program then separated out the vessels using an automated definitive threshold and divided the ONH and choroid into the vessel area and the capillary area. The following indices were calculated from the obtained data: mean blur rate (MBR, which reflects the OBF velocity), blowout time (BOT), and blowout score (BOS). These variables were analyzed separately in the ONH tissue (Tissue), in the ONH vessels (Vessel), throughout the ONH (All), and in the choroid (ChBFlow). BOS and BOT have previously been proposed as OBF profiles for evaluating vascular flexibility.

MBR is a measure of relative blood flow velocity and is expressed in arbitrary units (AU). It is calculated from the speckle pattern in the LSFG produced by the interference of laser light scattered by moving red blood cells in the ocular blood vessels.

BOT represents the proportion of time that the waveform is higher than half of the mean of the minimum and maximum signals during one heartbeat. A high BOT indicates that a high blood flow is maintained for a longer time during one heartbeat, indicating greater nutrition being supplied to the periphery.

BOS is another value developed to evaluate the amount of blood flowing through a blood vessel during one heartbeat. BOS is an index of the blood flow that is maintained between heartbeats and is calculated from the difference between the maximum and minimum MBRs as well as the average MBR. A high BOS indicates a high constancy of blood flow during the heartbeat, and is related to vascular resistance [18].

#### Systemic hemodynamics and ophthalmic examinations

The systolic blood pressure (SBP), diastolic blood pressure (DBP), heart rate (HR), intraocular pressure (IOP), red blood cell (RBC) count, fasting blood glucose (FBG), and triglyceride (TG), high-density lipoprotein cholesterol (HDL), and low-density lipoprotein cholesterol (LDL) levels were measured and the blood analysis performed at Department of Health Care Center of the Japan Community Health Care Organization, Tokyo Kamata Medical Center.

The participants were also asked about their smoking status. Blood pressure was recorded as the mean of two measurements made using a commercial sphygmomanometer after the participants had been seated for 10 minutes. Height and body mass were measured with participants wearing light clothing without shoes. Waist circumference was measured by using a flexible inch tape. The Body Mass Index (BMI) was calculated as body mass (kg) / height^2^ (m).

IOP was measured using a commercial sphygmomanometer (NIDEK, Aichi, Japan). The mean arterial pressure (MAP) was calculated as DBP + 1/3(SBP–DBP). The mean ocular perfusion pressure (OPP) was calculated as 2/3(MAP–IOP) [19]. This formula is based on evidence that the pressure in choroidal veins almost equals IOP [20].

#### Questionnaire on exercise habits

A questionnaire was used to estimate the presence of exercise habits, based on the frequency of exercise per week, duration of each exercise session, exercise intensity, exercise history, step per day and exercise types (Supporting information).

### Statistical analysis

Statistical analysis was performed using standard statistical software (version 25.0, SPSS Statistics, IBM Corporation, Armonk, NY, USA). Data are expressed as mean ± SD values. In all statistical analyses the cutoff for significance was 5%. ANOVA, the Kruskal-Wallis test, and the Mann-Whitney U test were used to compare normally distributed data. Spearman’s rank test was used to determine the coefficients for the correlations between the variables. Multiple logistic regression analysis was used to determine the associations between sex and the parameters of the pulse waveform analyses.

To examine the effects of aging and exercise habits, two-way ANOVA was used to evaluate the interactions of the OBF profile among groups. If a significant interaction is obtained, the two factors are considered to influence each other and hence cannot be separated. Bonferroni analysis was used as a post-hoc test to examine simple main effects.

Multiple linear regression analysis was used to identify which of the following factors independently affected the OBF profiles: sex, BMI, exercise habits, age squared, current smoking habit, IOP, HR, and the interaction of exercise habits and age squared.

## Results

The characteristics in the three age groups of this study are presented in Table 1. The height, body mass, BMI, waist circumference, RBC count, FBG, TG, LDL, and current smoking rate differed significantly among the three age groups. SBP and MAP were significantly higher in the elderly group, whereas DBP was significantly higher in the middle-aged group.

**Table 1:** Characteristics in the three age groups.

	Young (n = 140)	Middle-aged (n = 676)	Elderly (n = 64)	P
Age, years	37.5±2.7	50.6±6.3	69.5±3.7	<0.01*†‡
Height, cm	168.5±7.7	168.8±7.3	163.3±7.8	<0.01†‡
Body mass, kg	66.3±13.9	68.6±12.0	62.5±8.3	<0.01*‡
BMI, kg/m^2^	23.2±4.1	24.0±3.4	23.4±2.5	<0.01*
Waist circumference, cm	81.2±11.3	84.6±9.6	83.9±7.5	<0.01*†
SBP, mmHg	116.7±15.8	124.3±18.0	134.5±15.4	<0.01*†‡
DBP, mmHg	71.8±11.6	78.2±13.2	77.3±11.2	<0.01*†
MAP, mmHg	86.8±12.5	93.5±14.1	96.4±11.6	<0.01*†
HR, bpm	70.9±10.6	70.7±10.0	71.1±11.0	0.94
RBC, × 10^4^/μl	4.8±0.5	4.8±0.5	4.6±0.5	<0.01†‡
FBG, mg/dL	95.4±16.5	102.0±15.8	110.7±20.0	<0.01*†‡
TG, mg/dL	108.9±78.2	131.5±99.8	114.2±61.7	0.02*
HDL, mg/dL	63.3±17.6	63.6±17.1	64.8±16.7	0.75
LDL, mg/dL	120.0±30.5	131.6±31.5	133.1±26.9	<0.01*†
IOP, mmHg	11.9±2.4	12.0±2.7	11.9±2.8	0.96
OPP, mmHg	46.0±8.1	50.4±9.5	52.3±8.1	<0.01*†
MBR-All, au	26.1±4.0	25.4±4.5	23.4±5.0	<0.01†‡
BOS-All	81.4±3.6	80.7±4.1	73.9±5.1	<0.01†‡
BOT-All	54.9±3.8	52.2±3.7	47.9±3.8	0.01*†‡
MBR-Tissue, au	13.0±2.4	13.0±2.5	12.8±3.1	0.83
BOS-Tissue	78.9±3.7	77.8±4.4	70.3±5.8	<0.01*†‡
BOT-Tissue	52.2±4.2	49.3±3.8	44.9±3.7	<0.01*†‡
MBR-Vessel, au	46.0±7.3	45.4±7.0	42.6±7.4	<0.01†‡
BOS-Vessel	82.4±3.7	81.9±4.1	75.8±4.8	<0.01†‡
BOT-Vessel	56.0±3.8	53.8±3.9	49.8±3.8	<0.01*†‡
MBR-ChBFlow, au	9.7±3.1	9.2±3.0	9.5±3.5	0.19
BOS-ChBFlow	78.6±4.0	76.9±5.0	69.8±6.0	<0.01*†‡
BOT-ChBFlow	51.3±4.2	48.4±3.9	44.5±3.2	<0.01*†‡
Step per day	5663±3005	6697±3667	5700±3491	0.02*‡
Exercise intensity	5.9±2.2	5.4±2.2	4.4±1.9	0.02†
Current smoking (%)	39 (27.9)	219 (32.4)	9 (14.1)	0.01*†‡

Data are presented as mean ± SE.

P-value was come from one-way analysis of variance (one-way ANOVA) and Kruskal-Wallis test.

* Indicates statistically significant difference between the young and the middle-aged;

^†^ indicates statistically significant difference between the young and the elderly;

^‡^ indicates statistically significant difference between the middle-aged and the elderly (Dunnet test).

MBR-All, MBR-Vessel, and all sections of the BOS and BOT were significantly lower in the elderly groups. OPP was significantly higher in the elderly group. The number of steps per day was significant larger in the middle-aged group, while the average exercise intensity decreased significantly as age increased.

The characteristics in the two groups of self-reported exercise habits are presented in Table 2. Those in the Ex group were significantly older (51.0±9.5 years) than those in the Non-ex group (49.2±8.9 years). The current smoking rate was significantly higher in the Non-ex group than in the Ex group. The HDL level was significantly higher while the TG level was significantly lower in the Ex group than in the Non-ex group.

**Table 2:** Characteristics categorized by the self-reported Ex and Non-ex groups.

	Ex group (n = 321)	Non-ex group (n = 559)	P
Age, years	51.0±9.5	49.2±9.0	<0.01
Height, cm	168.0±7.7	168.5±7.4	0.31
Body mass, kg	68.0±11.5	67.6±12.6	0.97
BMI, kg/m2	24.0±3.3	23.7±3.6	0.31
Waist circumference, cm	80.0±8.7	84.1±9.9	0.34
SBP, mmHg	124.8±16.8	123.3±17.5	0.18
DBP, mmHg	76.8±12.8	77.3±13.1	0.63
MAP, mmHg	92.8±14.7	92.6±13.9	0.82
HR, bpm	69.2±10.1	71.7±10.0	<0.01
RBC, × 10^4^/μl	4.8±0.4	4.8±0.5	0.47
FBG, mg/dL	101.8±15.6	101.5±17.2	0.78
TG, mg/dL	115.2±69.1	133.2±106.1	0.01
HDL, mg/dL	66.5±17.8	62.0±16.5	<0.01
LDL, mg/dL	127.9±29.1	131.0±32.5	0.22
IOP, mmHg	11.9±2.6	11.9±2.7	0.33
OPP, mmHg	50.0±9.8	49.8±9.2	0.78
MBR-All, au	24.9±4.5	25.6±4.5	0.04
BOS-All	79.8±4.5	80.6±4.5	0.03
BOT-All	51.9±4.0	52.6±4.1	0.01
MBR-Tissue, au	12.8±2.4	13.0±2.6	0.31
BOS-Tissue	76.8±5.0	77.8±4.8	0.03
BOT-Tissue	49.0±4.2	49.7±4.2	0.01
MBR-Vessel, au	44.9±7.1	45.5±7.2	0.24
BOS-Vessel	81.2±4.4	81.8±4.4	0.01
BOT-Vessel	53.4±4.1	54.1±4.1	0.01
MBR-ChBFlow, au	9.2±3.2	9.4±3.0	0.08
BOS-ChBFlow	76.0±5.4	77.0±5.3	0.02
BOT-ChBFlow	48.1±4.1	48.8±4.3	<0.01
Step per day	7359±3883	5975±3318	<0.01
Exercise intensity	5.4±2.2		
Current smoking (%)	72 (22.4)	195(34.9)	0.03

Data are presented as mean ± SE.

P-value was come from two-sample t-test and Mann Whitney U test.

MBR-All was significantly higher in the Non-ex group than in Ex group. All sections of the average BOS and BOT. IOP and OPP did not differ significantly between the two groups. The number of steps per day was significantly larger in the Ex group than in the Non-ex group. The exercise intensity in the Ex group was 5.4±2.2 (out of 10).

The characteristics in the two groups categorized based on the MHLW definition (n = 192 and 688 in the Ex and Non-ex groups, respectively) in this study are presented in Table 3. Those in the Ex group were significant older (52.0±9.8 years) than those in the Non-ex group (49.3±8.9 years). The current smoking rate was significantly higher in the Non-ex group than in the Ex group. The HDL level was significantly higher while the TG level was significantly lower in the Ex group than in the Non-ex group. The HR was significantly higher in the Non-ex group than in the Ex group.

**Table 3:** Characteristics categorized by the MHLW-based Ex and Non-ex groups.

	Ex group (n = 192)	Non-ex group (n = 688)	P
Age, years	52.0±9.8	49.3±8.9	0.01
Height, cm	167.6±7.8	168.5±7.5	0.17
Body mass, kg	67.8±11.7	67.8±12.4	0.59
BMI, kg/m^2^	24.1±3.4	23.8±3.5	0.16
Waist circumference, cm	83.6±8.8	84.2±9.7	0.86
SBP, mmHg	125.3±17.9	123.5±17.9	0.3
DBP, mmHg	77.2±12.1	77.1±13.2	0.96
MAP, mmHg	93.2±13.2	92.6±14.1	0.58
HR, bpm	68.1±9.8	71.5±10.1	<0.01
RBC, × 10^4^/μl	4.8±0.5	4.8±0.5	0.42
FBG, mg/dL	101.8±15.2	101.5±17.0	0.85
TG, mg/dL	109.8±64.3	131.7±101.6	0.04
HDL, mg/dL	69.5±19.3	62.0±16.2	<0.01
LDL, mg/dL	128.3±30.3	130.4±31.7	0.08
IOP, mmHg	12.1±2.6	11.9±2.7	0.64
OPP, mmHg	50.2±9.1	49.8±9.4	0.64
MBR-All, au	24.5±4.5	25.6±4.5	<0.01
BOS-All	79.6±4.6	80.5±4.4	0.01
BOT-All	51.4±4.3	52.6±4.0	<0.01
MBR-Tissue, au	12.7±2.5	13.0±2.5	0.09
BOS-Tissue	78.1±4.7	74.9±4.5	0.01
BOT-Tissue	48.6±4.4	49.7±4.1	<0.01
MBR-Vessel, au	43.8±6.6	45.7±7.2	<0.01
BOS-Vessel	81.0±4.3	81.7±4.4	0.04
BOT-Vessel	53.0±4.4	54.1±4.0	<0.01
MBR-ChBFlow, au	9.0±3.1	9.4±3.0	0.08
BOS-ChBFlow	75.7±5.6	76.9±5.3	<0.01
BOT-ChBFlow	47.7±4.4	48.7±4.2	<0.01
Step per day	8069±4199	6013±3287	<0.01
Exercise intensity	5.6±2.2		
Current smoking (%)	32 (16.7)	232 (34.2)	<0.01

Data are presented as mean ± SE.

P-value was come from two-sample t-test and Mann Whitney U test.

MBR-All, MBR-Vessel, BOS-All, BOS-Vessel, BOS-ChBFlow, and BOT in all sections were significantly higher in the Non-ex group than in the Ex group, while BOS-Tissue was significantly lower in the Non-ex group. The number of steps per day was significantly larger in the Ex group than in the Non-ex group. The exercise intensity in the Ex group was 5.6±2.2.

The characteristics of the 698 males and 182 females in this study are shown in Table 4. The age of the male (50.1 ± 9.1 yrs) did not differ significantly from those of the female (49.1 ± 9.6 yrs). The height, body mass, BMI, waist circumference, RBC count, FBG, TG and current smoking rate of the male were all significantly higher than those of the female. The HDL of the female was significantly higher than those of the male, whereas the LDL and TG in the male were significantly higher than those in the female. SBP, DBP and MAP of the male were significantly higher than those of the female.

**Table 4:** The characteristics of male and female participants.

	Male (n = 698)	Female (n = 182)	P
Age, years	50.1±9.1	49.1±9.6	0.21
Height, cm	170.8±6.0	158.9±5.0	<0.01
Body mass, kg	70.7±11.0	56.6±10.0	<0.01
BMI, kg/m^2^	24.2±3.3	22.4±3.6	<0.01
Waist circumference, cm	85.4±8.9	78.8±9.8	<0.01
SBP, mmHg	125.3±17.8	118.3±17.4	<0.01
DBP, mmHg	78.9±12.6	70.0±12.0	<0.01
MAP, mmHg	94.4±13.7	86.1±12.8	<0.01
HR, bpm	70.9±10.3	70.5±9.4	0.71
RBC, × 10^4^/μl	4.9±0.4	4.4±0.4	<0.01
FBG, mg/dL	103.4±17.5	94.6±9.8	<0.01
TG, mg/dL	136.2±101.6	90.0±45.8	<0.01
HDL, mg/dL	60.5±15.3	75.8±18.2	<0.01
LDL, mg/dL	131.7±30.4	122.9±33.9	<0.01
IOP, mmHg	12.0±2.7	11.7±2.6	0.11
OPP, mmHg	50.9±9.2	45.7±8.7	<0.01
MBR-All, au	24.8±4.5	27.4±3.9	<0.01
BOS-All	80.9±4.3	77.9±4.4	<0.01
BOT-All	52.6±4.0	51.5±4.1	0.01
MBR-Tissue, au	12.8±2.6	13.6±2.3	<0.01
BOS-Tissue	78.1±4.7	74.9±4.5	<0.01
BOT-Tissue	49.7±4.2	48.5±4.0	<0.01
MBR-Vessel, au	44.6±7.0	48.2±6.9	<0.01
BOS-Vessel	82.1±4.2	79.1±4.3	<0.01
BOT-Vessel	54.1±4.1	53.1±4.0	<0.01
MBR-ChBFlow, au	9.4±3.1	9.2±3.1	0.62
BOS-ChBFlow	77.2±5.4	74.7±4.8	<0.01
BOT-ChBFlow	48.7±4.3	47.9±3.9	0.01
Step per day	6567±3718	6079±2977	0.01
Exercise intensity	5.5±2.2	4.8±2.0	0.42
Current smoking (%)	238(34.1)	29(15.9)	0.02

Data are presented as mean ± SE.

P-value was come from two-sample t-test and Mann Whitney U test.

The MBR-All, MBR-Tissue and MBR-Vessel in the male were significantly lower than those in the female. All sections of the BOS and BOT in the male were significantly higher than those of the female. The exercise intensity of the male was significantly higher than that of the female.

**Fig 1:**
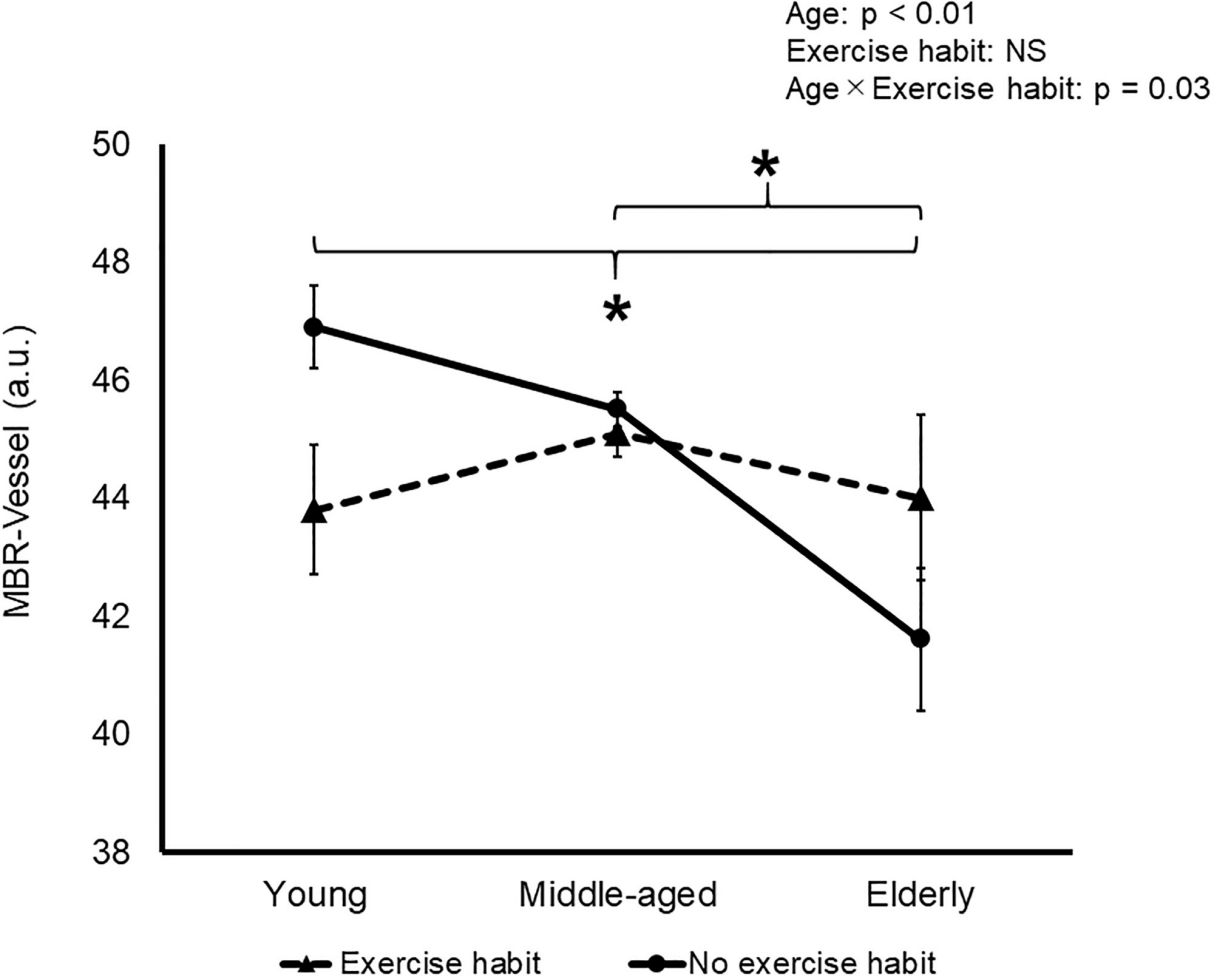
Effects of aging and self-reported exercise habits on MBR-Vessel. *, significant difference among young (<40 years old), middle-aged (41–64) and elderly (>65) groups without exercise habits; †, significant difference between groups without exercise habits. Data are mean and SD.

There was no significant interaction of age and exercise habits on IOP, OPP, MBR-All, MBR-Tissue, MBR-Choroid, or BOT and BOS in all sections. There was a significant interaction of age and exercise habits on MBR-Vessel, significantly decreasing in the Non-ex group among all three age groups (Fig 1).

**Fig 2:**
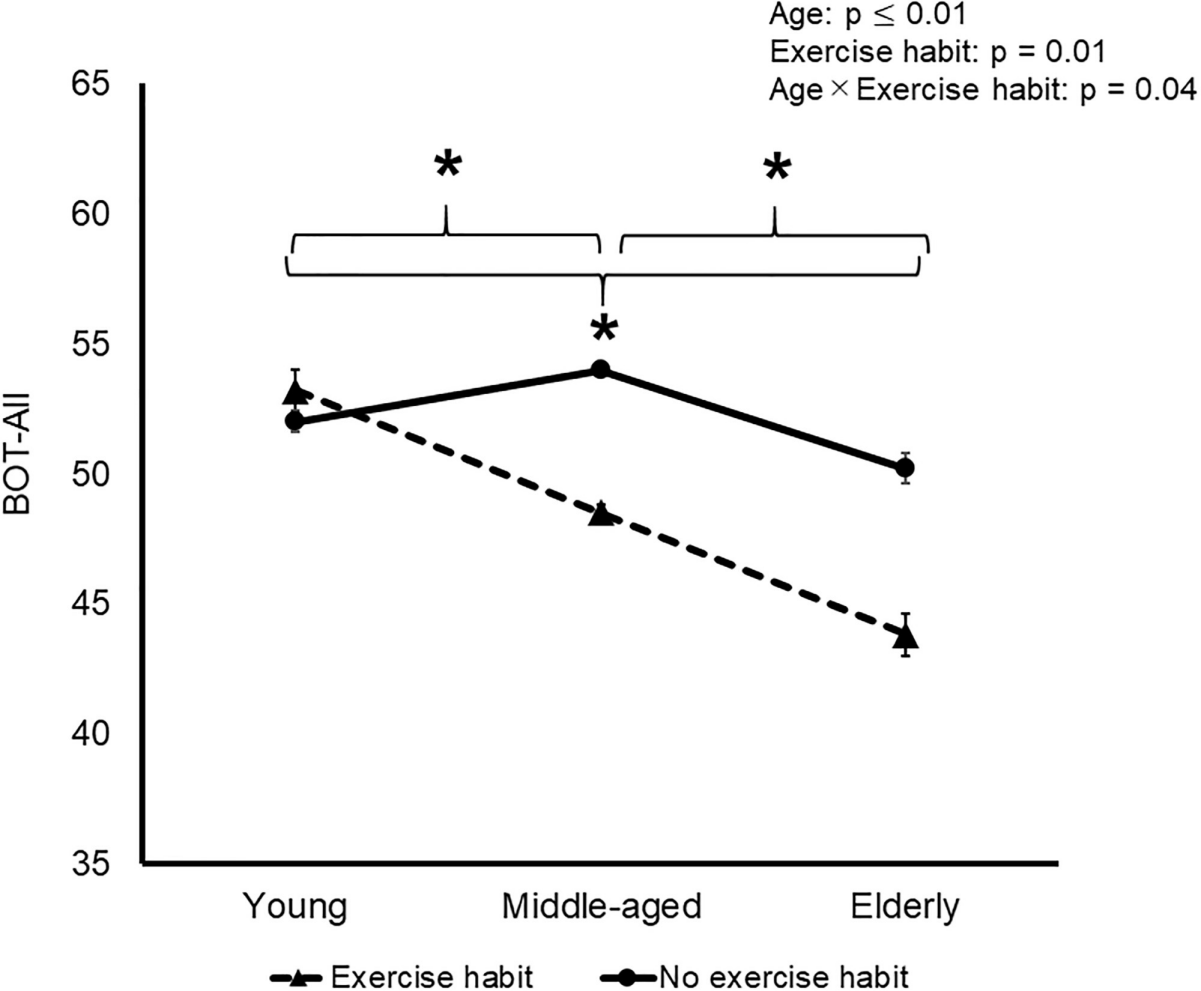
Effects of aging and MHLW-based exercise habits on BOT-All. *, significant difference among young (<40 years old), middle-aged (41–64) and elderly (>65) groups with and without exercise habits. age group. Data are mean and SD.

There was a significant interaction of age and exercise habits on BOT-All, which decreased significantly in the Ex group among all three age groups (Fig 2). There was no significant interaction of age and MHLW-based exercise habits on IOP, OPP, BOT-Vessel, or MBR and BOS in all sections. BOT-All in the Non-ex group was significantly higher in the middle-aged group.

**Fig 3:**
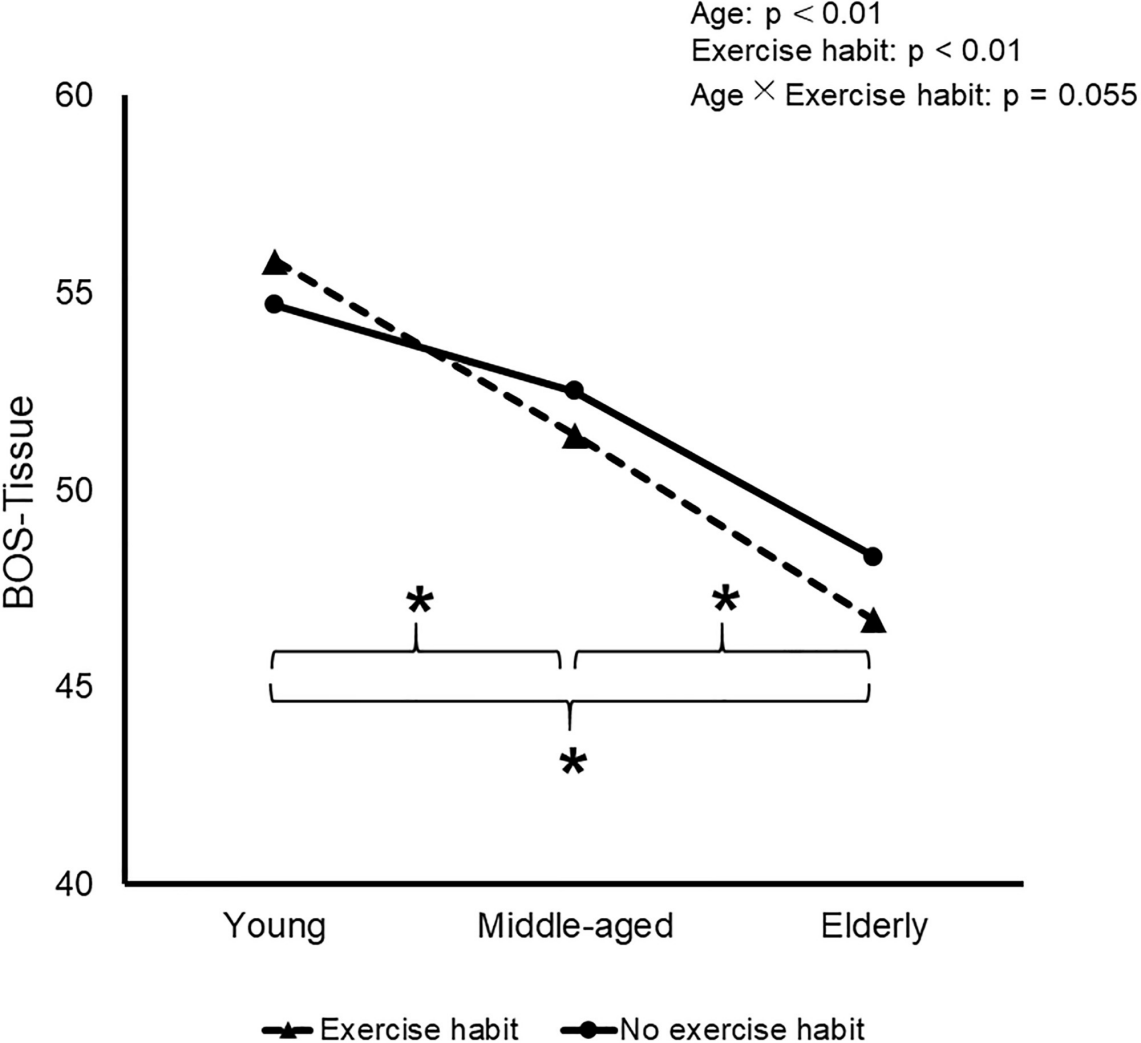
Effects of aging and MHLW-based exercise habits on BOS-Tissue. *, significant difference among young (<40 years old), middle-aged (41–64) and elderly (>65) groups with and without exercise habits. Data are mean and SD.

There tended to be significant interaction of age and exercise habits on BOS-Tissue. BOS-Tissue decreased significantly with age in both the Ex and Non-ex groups (p = 0.055, Fig 3).

Table 5 presents the standardized-coefficient and R-squared values for the systemic hemodynamics, ophthalmic observations, and exercise habits obtained in a multiple linear regression analysis. We determined the best regression formula for fitting the data on sex, BMI, self-reported exercise habits, age squared, current smoking, IOP, HR, exercise habits × age squared, and steps per day.

**Table 5:** Results of the multiple regression analysis for factors independently contributing to the item of ocular microcirculation, using the self-reported exercise habits.

	R^2^	Constant	Exercise habit	Steps per day	Sex(M:0,F;1)	Age^2^	BMI	Current smoking	IOP	Exercise habit*Age^2^	HR
**MBR-Vessel**	0.044	45.338	-2.001*	0.329	-0.864**	-1.521***	-0.317	0.453	0.010	1.759†	-0.032
**MBR-Tissue**	0.027	12.964	-0.026	0.017	-0.159	-0.015	-0.404***	0.101	0.311**	-0.012	-0.036
**MBR-All**	0.068	25.367	-0.914	0.017	-0.760***	-0.826***	-0.540**	0.228	0.125	0.771	0.088
**BOS-Vessel**	0.478	81.578	0.628	0.113	1.309***	-1.579***	0.306*	0.035	-0.507***	-0.690	2.039***
**BOS-Tissue**	0.559	77.551	0.958*	0.140	1.458***	-2.075***	0.178	-0.134	-0.388†	-1.215**	2.366***
**BOS-All**	0.529	80.360	0.708†	0.132	1.325***	-1.795***	0.271†	-0.015	-0.489***	-0.837*	2.165***
**BOT-Vessel**	0.330	53.860	0.070	-0.053	0.488**	-1.669***	0.034	0.156	-0.293*	-0.266	1.284***
**BOT-Tissue**	0.460	49.552	0.466	-0.107	0.672***	-2.000***	-0.229	-0.048	-0.097	-0.488	1.692***
**BOT-All**	0.429	52.414	0.115	-0.038	0.532***	-1.894***	-0.039	0.051	-0.200	-0.255	1.489***
**MBR-ChBFflow**	0.023	9.346	-1.152**	0.155	0.073	-0.302†	-0.417**	-0.107	0.141	0.982*	0.058
**BOS-ChBFflow**	0.460	76.813	0.528	0.180	1.162***	-2.263***	-0.077	0.013	-0.475**	-0.674	2.556***
**BOT-ChBFflow**	0.419	48.555	0.526	0.125	0.548***	-1.717***	-0.226	-0.042	-0.299*	-0.662	1.739***

*: p<0.05;

**: p<0.01;

***: p<0.001;

^†^: 0.05 < p < 0.08

Table 6 presents the standardized-coefficient and R-squared values for systemic hemodynamics, ophthalmic observations, and exercise habits obtained in a multiple linear regression analysis. We determined the best regression formula for fitting the data on sex, BMI, MHLW-based exercise habits, age squared, current smoking, IOP, exercise habits × age squared, and steps per day.

**Table 6:** Results of the multiple regression analysis for factors independently contributing to the item of ocular microcirculation, using the MHLW-based exercise habit.

	R^2^	Constant	Exercise habit	Steps per day	Sex(M:0,F;1)	Age^2^	BMI	Current smoking	IOP	Exercise habit*Age^2^	HR
**MBR-Vessel**	0.050	45.342	-2.382*	0.409	-0.797*	-1.418***	-0.326	0.282	-0.031	1.763†	0.013
**MBR-Tissue**	0.026	12.976	-0.153	0.018	-0.153	-0.051	-0.408***	0.088	0.295**	0.114	-0.025
**MBR-All**	0.068	25.386	-0.881	0.023	-0.745***	-0.797***	-0.560**	0.181	0.095	0.735	0.129
**BOS-Vessel**	0.479	81.616	0.798†	0.124	1.300***	-1.590***	0.305*	0.064	-0.535***	-0.780†	2.052***
**BOS-Tissue**	0.558	77.596	1.364**	0.123	1.439***	-2.128***	0.169	-0.073	-0.409**	-1.378**	2.377***
**BOS-All**	0.529	80.401	0.997*	0.131	1.311***	-1.814***	†0.266†	0.026	-0.515***	-0.979*	2.179***
**BOT-Vessel**	0.331	53.842	0.008	-0.017	0.471**	-1.650***	0.057	0.154	-0.280†	-0.362	1.247***
**BOT-Tissue**	0.453	49.525	0.214	-0.089	0.665***	-2.041***	-0.211	-0.039	-0.071	-0.327	1.653***
**BOT-All**	0.427	52.393	-0.058	-0.006	0.519***	-1.893***	-0.018	0.047	-0.185	-0.223	1.456***
**MBR-ChBFflow**	0.012	9.365	-0.675	0.116	0.058	-0.228	-0.450**	-0.102	0.125	0.687	0.103
**BOS-ChBFflow**	0.456	76.846	0.504	0.171	1.158***	-2.336***	-0.076	0.037	-0.503**	-0.487	2.570***
**BOT-ChBFflow**	0.412	48.537	0.179	0.130	0.547***	-1.814***	-0.211	-0.039	-0.281*	-0.341	1.708***

*: p<0.05;

**: p<0.01;

***: p<0.001;

^†^: 0.05 < p < 0.08

## Discussion

We have investigated the effects of aging and exercise habits on blood flow profiles throughout the ONH and choroidal arteries in a large population of participants in yearly medical checkups. The main findings of the present cross-sectional study were as follows: (1) an effect of aging on ocular circulation was demonstrated in a large population, supporting previous studies, (2) beneficial effects of exercise habits on ocular circulation were not supported; in contrast to our hypothesis, the ocular flow and profiles showed trends of vessel stiffening in the Ex group, and (3) ocular vessels were stiffer in males than in females.

BOT and BOS in various areas of the ocular circulation decrease with aging, regardless of the type of ocular vasculature. BOT and BOS throughout the ONH (i.e., All, Vessel, and Tissue) and the choroidal artery decreased with age, in accordance to several reports of vascular vessels throughout the ONH stiffening with age [18, 21, 22]. These results confirm the effects of age on vessel stiffness in the ocular circulation.

The present study found no preferable OBF profiles in the Ex group compared with the Non-ex group when exercise habits were assessed either in a self-reported manner or according to the MHLW definition. In short, exercise habits do not appear to improve the ocular circulation. A lack of exercise habits greatly influences the risk of vessel stiffening [23], whereas exercise habits improve the stiffness not only in limbs but also in many other regions of the body [4]. There are various mechanisms underlying how exercise habits can reduce the risks of stiffening and cerebrovascular diseases [24]. The TG and HDL levels, which are risk factors that exercise habits can improve, were lower in the Ex group. Nevertheless, exercise habits were not correlated with improved a blood flow profile in ocular vessels.

The effects of exercise habits on human arterial stiffness vary according to the site and size of the arteries [25]. Many previous studies have showed that exercise habits can reduce central arterial stiffness [26], especially that of large central arteries [25]. Exercising four or five times weekly is associated with reduced central arterial stiffness in older people. Vascular stiffness appears to be lowest in elderly who perform habitual physical activity of at least around 6,600 steps/day and/or spend more than 16 minutes/day performing exercise at an intensity of >3 metabolic equivalents [27]. Casual exercise training of two or three times weekly may be sufficient to minimize the stiffening of middle-sized arteries such as the carotid that is associated with aging [25]. However, exercise habits have only minor effects on small peripheral arteries [25], age-related decreases in basal limb blood flow and vascular conductivity [28], and chronically elevated arterial pressure [29, 30]. In contrast to large and central arteries and middle-sized arteries, the present study found a rather nonpreferable effect of exercise on BOT, as shown by an interaction in BOT-All and a marginally significant interaction (p = 0.055) in BOS-Tissue of age and MHLW-based exercise habits. BOS-Tissue and BOT-All of the elderly were lower in the Ex group than in the Non-ex group. The results suggest that the stiffness of ocular vessels cannot be improved by exercise habits.

The self-reported exercise intensity was roughly 5 (out of a maximum of 10), which can be considered moderate. Moderate levels of physical activity are associated with lower risks of vascular diseases in systemic regions compared to inactivity [31]. To our best knowledge, there is no explanation for the apparent increase in risk for moderate exercise associated with the stiffness of the ocular circulation. Moderate exercise habits cannot fully explain the lack of effect of exercise habits on the ocular circulation.

Exercise habits might have a marginal effect on ocular circulation. An interaction of age and self-reported exercise habits on ocular vessels was found in this study solely for MBR-Vessel. MBR-Vessel was the highest in middle-aged people with exercise habits. MBR, which is automatically calculated from variations in the degree of blurring, is a quantitative index of the blood flow, and measurements of MBR are highly reproducible [32–34]. MAP is well known to increase with age, which is consistent with the present data (Table 1). MBR increases when MAP has increased. It is therefore expected that MAP would increase with aging, with MBR consequently also increasing. MBR-Vessel did not differ between the Ex and Non-ex groups. It was inferred that there remains a possibility that MBR was counterbalanced against the increase in MAP with age, possibly via a decrease in the stiffness of the ocular vessels.

It was particularly interesting that MBR-Vessel and BOT-All peaked in the middle-aged participants. A further longitudinal study is necessary to identify the factors that restrict the effects of exercise habits on improving stiffness with age throughout the ONH.

MBR decreased during an acute increase in IOP in the ONH tissue [35]. Changes in IOP were found to be related to changes in BOS [36]. Those previous studies indicated that IOP is consistent with BOS and MBR in the ocular circulation, supporting our results.

BOS-Vessel and BOS-All in the ONH and choroid were correlated with BMI. BMI increases not only the risk of cardiovascular disease in people who are metabolically unhealthy [37], but also the risk of stiffening in the ocular circulation.

IOP did not differ with age, which is inconsistent with a negative correlation reported between IOP and age among Japanese healthy participants [38]. Studies in Europe and the US found a positive correlation between IOP and age [39, 40]. The different population compositions of these studies could explain these discrepant results. The present study included both healthy participants and those with metabolic syndrome. Some previous studies have suggested that five components—waist circumference, high TG, high blood pressure, high FBG, and low HDL—in the presence of metabolic syndrome each showed a positive correlation with IOP [41]; in short, these components can influence IOP.

### Limitations

To the best of our knowledge, this is the first study to have investigated the effects of exercise habits on OBF over a wide range of ages in a large population. Nevertheless, our study was subject to some limitations that need to be considered when interpreting the findings. Firstly, the study population comprised more males than females, sex may influence the ocular circulation [16] which has been reported. Sex imbalance might have affected the results in our study. We did not survey the use of oral contraceptives or hormone replacement therapy. These effects should not be ignored. Also, some of the participants were smokers. Smoking habits may influence the ocular circulation [42], though we included smoking habits and history of smoking in the analysis parameters. Similarly, the effects of alcohol consumption was not studied. Secondly, we did not determine the detailed exercise habits of the participants, and so the present findings need to be confirmed in a longitudinal study. The exercise habits were estimated by the questionnaire (see Supporting information) where participants recorded the exercise habits by themselves. The recording apparatus for step count was not controlled. Thirdly, we did not control for self-selection bias. Participants in a medical checkup program will be more health conscious than nonparticipants. Factors that probably affect the outcome of exercise habits were not assessed in the current study, such as dietary intake, the level of physical exercise in the Non-exercise group, social background, education level, and economic status. Fourthly, participants both in Ex and Non-ex groups had high fasting blood glucose (FBG). Blood flow in ophthalmic artery is decreased in patients with diabetes [43]. Fifthly, corneal thickness hasn’t been measured in this study. Corneal thickness influences IOP. As thicker corneas lead to higher IOP and thinner lead to lower of IOP [44].

## Conclusion

The present study investigated the effects of age and exercise habits on the ocular circulation by assessing BOS and BOT (which are indices of vascular function) as well as MBR in a large population. Effects of age on the ocular circulation were found, demonstrating the stiffening of ocular vessels with age. However, exercise habits did not alter the ocular circulation, while there were significant interactions of age and exercise habits on MBR-Vessel and BOT-All, and a marginal significant interaction on BOS-Tissue. These results suggest that the stiffness of ocular vessels increases with aging and that exercise habits cannot improve them. Therefore, the hypothesis that exercise habits can improve the deterioration in the stiffness of the ocular circulation with aging is rejected.

## Supporting information

S1 Questionnaire Questionnaire on exercise habits. (DOCX)

## References

[1] Wen SW, Wong CHY. Aging- and vascular-related pathologies. Microcirculation 2019;26(2):e12463. doi: 10.1111/micc.12463 29846990

[2] Ryskulova A, Turczyn K, Makuc DM, Cotch MF, Klein RJ, Janiszewski R. Self-reported age-related eye diseases and visual impairment in the United States: results of the 2002 National Health Interview Survey. Am J Public Health 2008;98(3):454–61. doi: 10.2105/AJPH.2006.098202 18235074 PMC2253577

[3] World Health Organization. Universal eye health: a global action plan 2014–2019. Spain: WHO Press 2019;1–19.

[4] Anderson A. J. Exercise and Glaucoma: Positive Steps Toward Finding Another Modifiable Risk Factor to Prevent Vision Loss. Ophthalmology 2019;126(7):965–66. doi: 10.1016/j.ophtha.2018.12.035 31229007

[5] Lee M. J, Wang J, Friedman D. S, Boland M. V, De Moraes C. G, Ramulu P. Y. Greater Physical Activity Is Associated with Slower Visual Field Loss in Glaucoma. Ophthalmology 2019;126(7):958–64. doi: 10.1016/j.ophtha.2018.10.012 30315900 PMC6458101

[6] Quigley C, Zgaga L, Vartsakis G, Fahy G. Refractive error and vision problems in children: association with increased sedentary behavior and reduced exercise in 9-year-old children in Ireland. JAAPOS 2019;23(3):159e1–e6. doi: 10.1016/j.jaapos.2018.12.011 31103561

[7] Green DJ. Exercise training as vascular medicine: direct impacts on the vasculature in humans. Exerc Sport Sci Rev 2009;37(4):196–202. doi: 10.1097/JES.0b013e3181b7b6e3 19955869

[8] Santos-Parker JR, LaRocca TJ, Seals DR. Aerobic exercise and other healthy lifestyle factors that influence vascular aging. Adv Physiol Educ 2014;38(4):296–307. doi: 10.1152/advan.00088.2014 25434012 PMC4315444

[9] Tanaka S. Status of physical activity in Japanese adults and children. Ann Hum Biol 2019;46(4):305–10. doi: 10.1080/03014460.2019.1635644 31234661

[10] Hayashi K, Sugawara J, Komine H, Maeda S, Yokoi T. Effects of aerobic exercise training on the stiffness of central and peripheral arteries in middle-aged sedentary men. Jpn J Physiol 2005;55(4):235–39. doi: 10.2170/jjphysiol.S2116 16248931

[11] Committee to Evaluate Diagnostic Standards for Metabolic Syndrome. Definition and the diagnostic standard for metabolic syndrome. Nihon Naika Gakkai Zasshi 2005;94:794–809. 15865013

[12] Teramoto T, Sasaki J, Ueshima H, Egusa G, Kinoshita M, Shimamoto K, et al. Metabolic syndrome. J Atheroscler Thromb 2008;15(1):1–5. doi: 10.5551/jat.e580 18319538

[13] Luft N, Wozniak PA, Aschinger GC, Fondi K, Bata AM, Werkmeister RM, et al. Ocular blood flow measurements in healthy white subjects using laser speckle flowgraphy. PLoS One 2016;11(12):e0168190. doi: 10.1371/journal.pone.0168190 27959905 PMC5154568

[14] Sugiyama T. Basic technology and clinical applications of the updated model of laser speckle flowgraphy to ocular diseases. Photonics 2014;1(3):220–34. doi: 10.3390/photonics1030220

[15] Wang L, Cull GA, Piper C, Burgoyne CF, Fortune B. Anterior and posterior optic nerve head blood flow in nonhuman primate experimental glaucoma model measured by laser speckle imaging technique and microsphere method. Invest Ophthalmol Vis Sci 2012;53(13):8303–09. doi: 10.1167/iovs.12-10911 23169886 PMC3525139

[16] Kobayashi T, Shiba T, Kinoshita A, Matsumoto T, Hori Y. The influences of gender and aging on optic nerve head microcirculation in healthy adults. Sci Rep 2019;9:15636. doi: 10.1038/s41598-019-52145-1 31666674 PMC6821724

[17] Yanagida K, Iwase T, Yamamoto K, Ra E, Kaneko H, Murotani K, et al. Sex-related differences in ocular blood flow of healthy subjects using laser speckle flowgraphy. Invest Ophthalmol Vis Sci 2015;56(8):4880–90. doi: 10.1167/iovs.15-16567 26225627

[18] Miyaji A, Ikemura T, Hayashi N. Effect of Aging on the Blowout Time in Various Ocular Vessels. J Aging Sci 2016;4(1):148. doi: 10.4172/2329-8847.1000148

[19] Robinson F, Riva CE, Grunwald JE, Petrig BL, Sinclair SH. Retinal blood flow autoregulation in response to an acute increase in blood pressure. Invest Ophthalmol Vis Sci 1986;27(5):722–726. 3700021

[20] Reitsamer HA, Kiel JW. A rabbit model to study orbital venous pressure, intraocular pressure, and ocular hemodynamics simultaneously. Invest Ophthalmol Vis Sci 2002;43(12):3728–34. 12454044

[21] Shiba T, Takahashi M, Matsumoto T, Shirai K, Hori Y. Arterial stiffness shown by the cardio-ankle vascular index is an important contributor to optic nerve head microcirculation. Graefes Arch Clin Exp Ophthalmol 2017;255(1):99–105. doi: 10.1007/s00417-016-3521-9 27743161 PMC5203816

[22] Shiga Y, Omodaka K, Kunikata H, Ryu M, Yokoyama Y, Tsuda S, et al. Waveform analysis of ocular blood flow and the early detection of normal tension glaucoma. Invest Ophthalmol Vis Sci 2013;54(12):7699–7706. doi: 10.1167/iovs.13-12930 24130177

[23] Kohl HW 3rd, Craig CL, Lambert EV, Inoue S, Alkandari JR, Leetongin G, et al. The pandemic of physical inactivity: global action for public health. Lancet 2012;380(9838):294–305. doi: 10.1016/S0140-6736(12)60898-8 22818941

[24] Anderson FA Jr, Spencer FA. Risk factors for venous thromboembolism. Circulation 2003;107(23):I9–16. doi: 10.1161/01.CIR.0000078469.07362.E6 12814980

[25] Shibata S, Fujimoto N, Hastings JL, Carrick-Ranson G, Bhella PS, Hearon CM Jr, et al. The effect of lifelong exercise frequency on arterial stiffness. J Physiol 2018;596(14):2783–2795. doi: 10.1113/JP275301 29781119 PMC6046080

[26] Ashor AW, Lara J, Siervo M, Celis-Morales C, Mathers JC. Effects of exercise modalities on arterial stiffness and wave reflection: a systematic review and meta-analysis of randomized controlled trials. PLoS One 2014;9(10):e110034. doi: 10.1371/journal.pone.0110034 25333969 PMC4198209

[27] Aoyagi Y, Park H, Kakiyama T, Park S, Yoshiuchi K, Shephard RJ. Yearlong physical activity and regional stiffness of arteries in older adults: the Nakanojo Study. Eur J Appl Physiol 2010;109(3):455–64. doi: 10.1007/s00421-010-1357-2 20145948

[28] Dinenno FA, Seals DR, DeSouza CA, Tanaka H. Age-related decreases in basal limb blood flow in humans: time course, determinants and habitual exercise effects. J Physiol 2001;531(Pt 2):573–79. doi: 10.1111/j.1469-7793.2001.0573i.x 11230528 PMC2278480

[29] Ferrier KE, Waddell TK, Gatzka CD, Cameron JD, Dart AM, Kingwell BA. Aerobic exercise training does not modify large-artery compliance in isolated systolic hypertension. Hypertension 2001;38(2):222–26. doi: 10.1161/01.hyp.38.2.222 11509480

[30] Seals DR, Tanaka H, Clevenger CM, Monahan KD, Reiling MJ, Hiatt WR, et al. Blood pressure reductions with exercise and sodium restriction in postmenopausal women with elevated systolic pressure: role of arterial stiffness. J Am Coll Cardiol 2001;38(2):506–13. doi: 10.1016/s0735-1097(01)01348-1 11499745

[31] Armstrong MEG, Green J, Reeves GK, Beral V, Cairns BJ, Million Women Study Collaborators. Frequent physical activity may not reduce vascular disease risk as much as moderate activity: large prospective study of women in the United Kingdom. Circulation 2015;131(8):721–29. doi: 10.1161/CIRCULATIONAHA.114.010296 25688148

[32] Takahashi H, Sugiyama T, Tokushige H, Maeno T, Nakazawa T, Ikeda T, et al. Comparison of CCD-equipped laser speckle flowgraphy with hydrogen gas clearance method in the measurement of optic nerve head microcirculation in rabbits. Exp Eye Res 2013;108:10–15. doi: 10.1016/j.exer.2012.12.003 23262066

[33] Aizawa N, Nitta F, Kunikata H, Sugiyama T, Ikeda T, Araie M, et al. Laser speckle and hydrogen gas clearance measurements of optic nerve circulation in albino and pigmented rabbits with or without optic disc atrophy. Invest Ophthalmol Vis Sci 2014; 55(12):7991–96. doi: 10.1167/iovs.14-15373 25377226

[34] Aizawa N, Yokoyama Y, Chiba N, Omodaka K, Yasuda M, Otomo T, et al. Reproducibility of retinal circulation measurements obtained using laser speckle flowgraphy-NAVI in patients with glaucoma. Clin Ophthalmol 2011;5:1171–76. doi: 10.2147/OPTH.S22093 21887100 PMC3162298

[35] Kiyota N, Shiga Y, Ichinohasama K, Yasuda M, Aizawa N, Omodaka K, et al. The impact of intraocular pressure elevation on optic nerve head and choroidal blood flow. Invest Ophthalmol Vis Sci 2018;59(8):3488–96. doi: 10.1167/iovs.18-23872 30025080

[36] Takeshima S, Higashide T, Kimura M, Udagawa S, Yamada Y, Takemoto D, et al. Effects of trabeculectomy on waveform changes of laser speckle flowgraphy in open angle glaucoma. Invest Ophthalmol Vis Sci 2019;60(2):677–84. doi: 10.1167/iovs.18-25694 30786279

[37] Haraguchi N, Koyama T, Kuriyama N, Ozaki E, Matsui D, Watanabe I, et al. Assessment of anthropometric indices other than BMI to evaluate arterial stiffness. Hypertens Res 2019;42(10):1599–1605. doi: 10.1038/s41440-019-0264-0 31019248

[38] Fukuoka S, Aihara M, Iwase A, Araie M. Intraocular pressure in an ophthalmologically normal Japanese population. Acta Ophthalmol 2008;86(4):434–39. doi: 10.1111/j.1600-0420.2007.01068.x 18039350

[39] Wu SY, Leske MC. Associations with intraocular pressure in the Barbados Eye Study. Arch Ophthalmol 1997;115(12):1572–76. doi: 10.1001/archopht.1997.01100160742012 9400792

[40] Klein BE, Klein R, Linton KL. Intraocular pressure in an American community. The Beaver Dam Eye Study. Invest Ophthalmol Vis Sci 1992;33(7):2224–28. 1607232

[41] Wang YX, Tao JX, Yao Y. The association of intraocular pressure with metabolic syndrome and its components: a meta-analysis and systematic review. Int J Ophthalmol 2019;12(3):510–16. doi: 10.18240/ijo.2019.03.24 30918823 PMC6423386

[42] Thornton J, Edwards R, Mitchell P, et al. Smoking and age-related macular degeneration: a review of association. Eye (Lond) 2005;19(9):935–44. doi: 10.1038/sj.eye.6701978 16151432

[43] Kraśnicki P, Mariak Z, Ustymowicz A, Proniewska-Skretek E. Assessment of blood flow in the ocular circulation in type 2 diabetes patients with Color Doppler imaging. Klin Oczna 2006;108(7–9):294–98. 17290827

[44] Rand R, Allingham KF, Damji S. Shield’s Textbook of Glaucoma. Fifth Indian edition. Lippincott Williams & Wilkins; 2005.

