# Supplementary material for: Effects of aging and exercise habits on blood flow profile of the ocular circulation
Source: PLoS One. 2022 Apr 14;17(4):e0266684. doi: 10.1371/journal.pone.0266684 (PMC9009706; doi:10.1371/journal.pone.0266684)
Supplement: S1 Questionnaire — (DOCX) [file pone.0266684.s001.docx]

Supporting Information

Questionnaire on exercise habits

Name: Sex: Male / Female Age:

1. Have you been exercising regularly over the past year?

Yes / No (if you answer no, please go to Question 6)

2. How often do you exercise?

( 　 ) times per week ( 　 ) minutes at a time

3. Which exercise do you perform often? Please write the two most frequent exercises.

For example: walking, cycling, gym, jogging, and running

( ), ( )

4. What is the intensity of your exercise? Please circle one number.

1 2 3 4 5 6 7 8 9 10

5. If you know, how many steps do you walk a day (activities such as jogging are included)

(　　　　　) steps per day

6. Exercise history. Which exercise(s) do you perform often? For how many years have you performed them?

In your twenties

_____________________________________________________________

In your thirties _____________________________________________________________

In your forties

_____________________________________________________________

In your fifties

_____________________________________________________________

In your sixties

_____________________________________________________________
